# P23 Cracking the case: CNS lymphoma and HIV through the lens of lab systems

**DOI:** 10.1093/jacamr/dlaf230.030

**Published:** 2025-12-04

**Authors:** Aqsa Mahreen, Bilal Mian, Aiden Plant

**Affiliations:** Dept of Microbiology, Walsall Healthcare NHS Trust, UK; Dept of Microbiology, Walsall Healthcare NHS Trust, UK; Dept of Microbiology, Black Country Pathology Services, The Royal Wolverhampton NHS Trust, UK

## Abstract

**Background:**

HIV-associated immunodeficiency is a risk factor for the development of primary CNS lymphoma; the clinical manifestations of which are often non-specific.^1^ Similarly, CNS ring enhancing lesions can be associated with a plethora of conditions including abscesses, toxoplasmosis, tuberculomas and primary CNS lymphomas.^2^

**Case presentation:**

A 61-year-old woman with a no significant medical history presented to the emergency department with a 1 day history of myotonic jerking of her right arm and leg, alongside 1 week of right-sided weakness, weight loss for the last 3 years and three episodes of shingles, most recently 3 months prior to admission. There was no exposure to animals, consumption of unpasteurized milks, TB contacts, use of illicit drugs, or any travel outside the UK in the last decade. On presentation she was cachectic; neurological findings were power on the MRC scale of 4/5 in the right upper and 2/5 in the right lower limb respectively. The remaining examination findings were unremarkable including for rashes or lymphadenopathy. Blood tests revealed a Hb 119 g/L, WCC 3.10 × 10^9^/L, platelet count 118 × 10^9^/L, lymphocyte count of 0.64 × 10^9^/L, whilst the remainder were unremarkable. All admissions are routinely screened for HIV and her serology was positive for HIV-1 with a viral load of 387 000 copies/mL, resulting in a new diagnosis of HIV. An initial CT brain scan showed no acute intracranial findings but given the report of seizure like activity the patient underwent an MRI scan of the brain and spine, which revealed a 2.2×2.4 cm ring enhancing lesion in left parietal convexity, with perifocal oedema and mass effect. Further diagnostic tests were performed to elucidate her diagnosis; the CD4 count was 27 cells/mm^3^. Toxoplasma IgM and IgG and syphilis antibodies were negative, both EBV and CMV revealed evidence of past infection with viral loads of 132 and 49 IU/mL in plasma respectively, cryptococcal antigen in serum was negative, as was BK, JC and HHV-8 DNA PCR. Lumbar puncture and CSF analysis showed a white blood cell count of one, with 54 red blood cells, a protein of 0.3 g/L and glucose of 4.2 mmol/L (serum 6.4), with no organisms seen nor cultured. Molecular testing on CSF returned negative for enterovirus, herpes virus, varicella virus, EBV, CMV, toxoplasma, BK, JC, HHV-8 and -6, Listeria monocytogenes, *Haemophilus influenzae*, *Neisseria meningitidis*, *Streptococcus pneumoniae* and *Mycobacterium tuberculosis* complex. In lieu of the above results the patient was transferred to the local tertiary centre for MR spectroscopy and brain biopsy, leading to a diagnosis of non-Hodgkin B-cell lymphoma, an AIDS defining illness, for which chemotherapy has been commenced.^3^

**Conclusions:**

This case highlights the importance of the laboratory systems in providing clarity. The effectiveness of the HIV screening programme was pivotal in this case, providing a focal point and bringing more niche diagnoses to the fore in a patient with nonspecific presentation. There is a possibility that without the prevalence of the screening programme there may have been a delay in diagnosis and treatment.
